# Potential therapeutic value of necroptosis inhibitor for the treatment of COVID-19

**DOI:** 10.1186/s40001-022-00913-7

**Published:** 2022-12-09

**Authors:** Yongan Kang, Qinghai Wang

**Affiliations:** 1 Cardiovascular Medicine, The Second People’s Hospital Affiliated to Three Gorges University/Yichang Second People’s Hospital, Yichang, 443000 China; 2 Three Gorges University College of Basic Medicine, Yichang, 443000 China; 3 Yichang Clinical Medical Research Center for Prevention and Treatment of Chronic Heart Failure, Yichang, 443000 China

**Keywords:** Necroptosis, Therapeutics, SARS-CoV2, COVID-19, Inhibitor

## Abstract

The coronavirus disease 2019 (COVID-19), caused by a novel virus of the beta-coronavirus genus (SARS-CoV-2), has spread rapidly, posing a significant threat to global health. There are currently no drugs available for effective treatment. Severe cases of COVID-19 are associated with hyperinflammation, also known as cytokine storm syndrome. The reduce inflammation are considered promising treatments for COVID-19. Necroptosis is a type of programmed necrosis involved in immune response to viral infection, and severe inflammatory injury. Inhibition of necroptosis is pivotal in preventing associated inflammatory responses. The expression of key regulators of the necroptosis pathway is generally up-regulated in COVID-19, indicating that the necroptosis pathway is activated. Thus, necroptosis inhibitors are expected to be novel therapeutic candidates for the treatment of COVID-19.

Better knowledge of the necroptosis pathway mechanism is urgently required to solve the remaining mysteries surrounding the role of necroptosis in COVID-19. In this review, we briefly introduce the pathogenesis of necroptosis, the relationship between necroptosis, cytokine storm, and COVID-19 also summarizes the progress of inhibitors of necroptosis. This research provides a timely and necessary suggest of the development of necroptosis inhibitors to treat COVID-19 and clinical transformation of inhibitors of necroptosis.

## Introduction

Necroptosis (programmed necrosis) is an emerging form of programmed cell death, which is involved in development, immune response to viral infection, and inflammatory injury [1, 2]. Necroptosis is marked by rupture of the plasma membrane and release of proinflammatory damage-associated molecular patterns (DAMPs) that mediate cell death [3]. The cells release danger signals DAMPs, which act as activators and amplifiers of the inflammatory response [4]. DAMPs are recognized by a series of receptors called “pattern recognition receptors,” such as Toll-like Receptors (TLRs) and receptors for advanced glycation end products, which activate the innate immunity and further evoke the release of cytokines. The release of cytokines, in turn, induces more necrosis and triggers an inflammatory cascade that causes Cytokine storm syndromes (CSS) [4]. CSS are characterized by rampant and often fatal systemic hyperinflammation. Accumulating evidence indicates that many individuals with severe Coronavirus disease-19 (COVID-19) exhibit cytokine storm syndrome. As early as 2020, a new edition of anti-tumor necrosis factor therapy (TNF-α) for COVID-19 has been proposed by scholars [5].

COVID-19 is caused by SARS Coronavirus-2 (SARS-CoV-2) infection. The typical symptoms of COVID-19 include fever, sore throat, fatigue, cough, and dyspnea combined with recent exposure. This new type of coronavirus is quickly spreading throughout the world and placing a heavy burden on global health care systems. Currently, the Centers for Disease Control, together with the SARS‐CoV‐2 interagency committee, have identified three categories of SARS‐CoV‐2 variants: Variants of interest (VOI), variants of concern (VOC), and variants of high consequence (VOHC), which include the widely popular Alpha, Beta, Gamma, Epsilon, Delta, and Omicron variant. These virus variants have a higher transmission rate and can lead to a substantial decrease in treatment potency and vaccine performance [6]. The newly discovered B.1.617.2\Delta variant and B.1.1.529\Omicron variant may affect clinical outcomes by reducing T cell reactivity [7]. Based on this, SARS-CoV-2 Assessment of Viral Evolution (SAVE) has been launched. The purpose of this program is to assess the impact of SARS-CoV2 variants on diagnosis, vaccine and treatment, as well as potential public health risks. As of November 23, 2022, 3 years after the discovery of the human coronavirus SARS-CoV-2, the World Health Organization reported globally over 634 million confirmed cases and 6.60 million deaths from COVID-19 (World Health Organization, 2021). To date, no effective drug treatment is available for fighting this COVID-19. Cytokine storm is a marker of COVID-19 illness severity and increased mortality. To reverse the deterioration of severe and critically ill patients from this disease, blocking cytokine storm has become a critical therapeutic target [8]. Animal experiments show that necroptosis inhibitors can reduce the release of inflammatory factors and save tissue damage [9].

In this review, we briefly introduce the pathogenesis of necroptosis, and the relationship between necroptosis, cytokine storm and COVID-19 also summarizes the progress of inhibitors of necroptosis. Finally, we propose that targeting necroptosis provides a potential strategy for COVID-19 treatment. We expect this review to provide researchers with valuable ideas and approaches in quickly finding out the precise therapies and effective drugs against COVID-19.

### Molecular mechanism of necroptosis

**Fig. 1:**
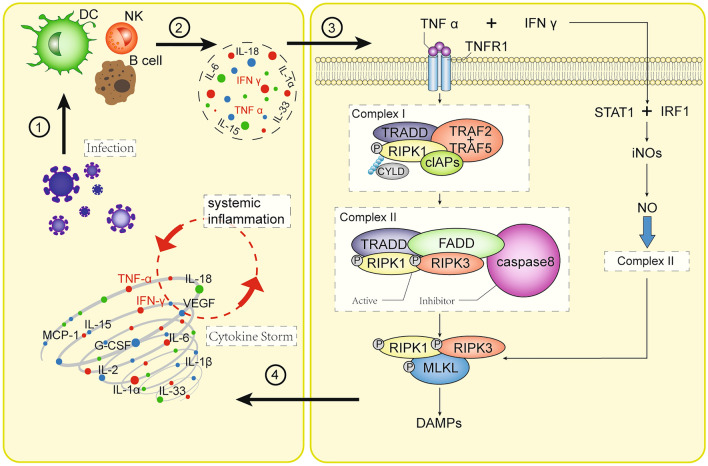
Programmed necrosis by virus infection (1) (1) The human immune system is activated in response to viral infections; (2) immune cells release cytokines, including TNF-α; (3) TNF-α complementary receptor, leading to formation of short-lived membrane signaling complex (known as complex I) and complex . Eventually, leads to DAMP release from cell; (4) As a result, the “cytokine storm” pathology is exacerbated. (2) TNF-α can stimulate together with IFN-γ on the STAT1 and IRF1 pathways to enhance the necroptosis

Necroptosis is a form of non-apoptotic cell death playing important roles in many inflammatory conditions and related diseases [1]. Much of what we know about necroptosis signaling mechanisms comes from the study of cell death induced by the proinflammatory cytokine TNF. TNF-α-mediated necroptosis is a classical necroptosis which binds with complementary receptor leading to formation of short-lived membrane signaling complex (known as complex I), including Receptor-interacting protein kinase 1 (RIPK1), TNFR-associated death domain (TRADD), cellular inhibitor of apoptosis protein 1 (cIAP1), cIAP2, TNFR-associated factor 2 (TRAF2), and TRAF5 [10]. RIPK1 is a crucial regulator of cell fate in complex I [11]. RIPK1 is ubiquitination by cIAP1/2 and TRAF2/5 which resulted in formation of stable complex I and initiate alternative pathway that culminates with cell survival pathway, including NF-кB and MAPK-mediated pathway [12]. RIPK1 is deubiquitinated by the deubiquitinase cylindromatosis (CYLD), which subsequently limits the sustained activation of NF-κB signaling [13] and leads to a tendency toward the activation of cell death pathways. Removal of ubiquitin chain from RIPK1 leads to its interaction with FADD, TRADD, RIPK3, and caspase-8 which further resulted in formation of complex II. Complex II is involved in the activation of both apoptosis and necroptosis pathways. In complex II, active caspase-8 cleaves both RIPK1 and RIPK3, resulting in their inactivation, and the proapoptotic caspase activation cascade is initiated, ultimately leading to apoptosis execution [14]. However, following the inhibition of caspase-8 due to pharmaceutical or genetic intervention [10], RIP kinases cleavage stops, and the cell death pathway is directed to necroptosis. RIPK1 interacts with RIPK3 through receptor homology domain (RHD) leading to formation of necrosome which further initiates the downstream signaling resulting in necroptosis [15]. The ultimate execution step consists of mixed lineage kinase domain-like protein (MLKL) phosphorylation by RIPK3, triggering MLKL oligomerization, which is indispensable for its translocation from the cytosol to the plasma membrane [16, 17]. MLKL-dependent necroptotic membrane permeabilization subsequently induces release of cellular cytoplasmic material and specific DAMPs that can promote inflammation in distal tissues [18–20] (Fig. 1).

### Cytokine storm, COVID-19, and necroptosis

SARS-CoV-2 activates innate and acquired immune response, and further impairs the immune system and causes cytokine storm [21]. Serum cytokine levels that are elevated in patients with COVID-19-associated cytokine storm include TNF-α and IFN-γ. On one hand, TNF-α can mediate the occurrence of necroptosis, and on the other hand, it can stimulate together with IFN-γ on the STAT1 and IRF1 pathways to produce inducible NO synthase (iNOS) that is the enzyme responsible for production of NO [22]. NO can interact with the caspase-8 and FADD in complex II to enhance the necroptosis. Necroptosis is marked by rupture of the plasma membrane and release of DAMPs [3]. Injured cells release danger signals DAMPs, which act as activators and amplifiers of the inflammatory response [4]. DAMPs are recognized by a series of receptors called “pattern recognition receptors,” such as TLRs and receptors for advanced glycation end products, which activate the innate immunity and further evoke the release of cytokines [4]. The release of these inflammatory cytokines triggers an inflammatory cascade which leads to the systemic inflammatory response syndrome.

The physical effect of the cytokine storm is damage from epithelial injury, increased capillary permeability, and organ dysfunction. Acute respiratory distress syndrome (ARDS) caused by cytokine storm is the main mortality factor in COVID-19. Inhibition of necroptosis with necrostatin-1 or genetic deletion of RIPK3 improves outcomes in mouse models of ARDS [23]. Moreover, sepsis was also observed as a common complication during COVID-19 [24]. Studies on Ripk3-deficient mice showed protective effects in models of TNF-induced and sepsis [25].

Necroptosis markers, including RIPK1, RIPK3 and MLKL, have been detected in both human and animal models of the COVID-19 infection. Cytokine production contributes to the activation of platelets. Increased platelet activation and platelet–monocyte aggregate formation were present in severe COVID-19 patients, but not in patients with mild self-limiting COVID-19 syndrome [26]. Immunofluorescence of blood of patients with COVID-19 showed the presence of p-MLKL in platelets that stained positive for SARS-CoV-2 spike protein [27]. RIPK1 activation was found in the upper respiratory epithelial cells of COVID-19 patients [9]. At the same time, it was reported that the median level of serum RIPK3 was higher in severe patients compared to mild ones [28]. In the hamster, both organotypic cultures, consistent with the uncontrolled expression of TNF-α and high levels of MLKL mRNA, found over the four days of infection in both the lungs and brainstem, compared to non-infected cultures [29]. The above studies indicate that the necroptosis pathway is activated in COVID-19.

### Classification of necroptosis inhibitor

Currently, RIPK1, RIPK3, and MLKL have been widely recognized as critical therapeutic targets of the necroptosis machinery. Targeting RIPK1, RIPK3, and/or MLKL is a promising strategy for necroptosis-related diseases.

Necrostatin-1 s (Nec-1 s) was the first small-molecule inhibitor of RIPK1 kinase to be developed and has been widely used to investigate the role of RIPK1 in mechanistic studies and animal models of human diseases [29–33]. Although Nec-1 is a good research tool to investigate the involvement of RIPK1 kinase activity in experimental disease models, it shows moderate potency [34], poor pharmacokinetics (PK) properties [31, 35], and moderate specificity due to off-target effects on indoleamine-2,3-dioxygenase (IDO)[32, 36], which may affect the immune system, especially regulatory T cells [37]. This poor PK profile of Necs-1 s limits their further development for therapeutic use [34, 38]. From a high-throughput screening of GSK DNA-encoded libraries [39], a new class of benzoxazepinone (BOAs) or GSK’841-based RIPK1 inhibitors was identified [38, 40]. These inhibitors, especially the optimized inhibitor GSK2982772, are superior to other designed RIPK1 inhibitors due to their high potency, selectivity, and better PK profiles [40]. A few small-molecule inhibitors of RIPK1 that offer high selectivity have been developed by different companies and have successfully entered phase I/II clinical trials in humans (source: ClinicalTrials.gov). RIPK1 inhibitor is currently being tested in human clinical trials for COVID-19 treatment (NCT04469621). In addition to the above RIPK1 inhibitory drugs under study, among the drugs currently in clinical use, the aromatic antiepileptic drug primidone has been found to be an effective inhibitor of RIPK1 [9], which mainly inhibits the interaction between RIPK1 and FADD. By preventing the initial formation of complex II, a key step in necroptosis is blocked. A clinical study of primidone in the treatment of COVID-19 by blocking RIPK1 is currently under application to the European Union’s Drug Regulatory Agency Clinical Trials Database (EudraCT).

Although RIPK3 is thought to be a more specific regulator of necroptosis than RIPK1, the translation of RIPK3 inhibitors to clinical applications has been lagging. Kaiser et al. discovered several compounds such as GSK’840, GSK’843, and GSK’872 that can bind to and inhibit RIPK3 with high potency [41]. However, when administered alone, these compounds induce apoptosis in a concentration-dependent manner [42], suggesting that it induces a RIPK3 proapoptotic conformation, which may complicate its development toward clinical use. Inhibition of RIPK3 kinase activity by dabrafenib does not appear to favor apoptosis, in contrast to the RIPK3 kinase inhibitors GSK’872 and GSK’843, which enhance RIPK1/FADD/CASP8 complex formation and apoptosis [42]. Interestingly, a group at GSK has identified a class of inhibitors, represented by GSK’067 and GSK’074, targeting both RIPK1 and RIPK3, which completely blocked necroptosis in both human and murine cells [43]. In contrast to GSK’872 and despite a structural resemblance, apoptosis was not induced by this inhibitor.

The above mainly focuses on the effect of directly inhibiting RIPK3 to prevent necroptosis. The complex of heat shock protein 90 (HSP90) and co-chaperone CDC37 can regulate the stability and function of RIP3 and MLKL, and play a key role in the activation of RIP3 [44]. The use of HSP90 inhibitors can also indirectly inhibit RIPK3-dependent necroptosis. Cun Li et al. found that three highly pathogenic human coronaviruses, including SARS-CoV-2, are highly dependent on Hsp90-dominated cellular chaperone proteins, and they require the help of these chaperone proteins to fold correctly to ensure structure and functional integrity, and when the Hsp90 inhibitor 17-AAG was used, the viral titer was significantly reduced with the occurrence of virion protein degradation [45]. The current in vitro study supports that an oral Hsp90 inhibitor SNX-5422 can inhibit the replication of SARS-CoV-2 with high selectivity and may prevent the early inflammatory factor storm. The specific mechanism needs to be further studied, and necroptosis cannot be ruled out [46]. In addition to the above HSP90 inhibitors, kongensin A (KA), a natural product isolated from croton, also uses HSP90 as a direct target and can effectively inhibit RIP3-dependent necroptosis [47].

MLKL is the immediate downstream mediator of RIP3 in necroptosis. However, compared with RIPK1 and RIPK3, fewer inhibitors targeting MLKL have been discovered. Necrosulfonamide (NSA), a well-known MLKL inhibitor, inhibits necroptosis by blocking human MLKL phosphorylation, but not the rodent homolog, thus invalidating pharmacological, pharmacokinetic and toxicity preclinical testing [48]. There are reports that the coiled-coil domain (CCD) of Beclin 1 binds to the CCD of MLKL, which restrains the oligomerization of phosphorylated MLKL. These results suggest that Beclin 1 functions as a negative regulator in the execution of necroptosis by suppressing MLKL oligomerization [49].

## Conclusion and perspectives

Necroptosis is a necrotic form of programmed cell death that is involved in various inflammatory pathologies [1, 50]. Increasing evidence suggests that necroptosis play an important role in COVID-19 pathophysiology. Through various drugs or compounds to inhibit necroptosis pathways may become a new method of immunotherapy for the COVID-19.

The expression of key regulators of the necroptosis pathway is generally up-regulated in COVID-19, indicating that the necroptosis pathway is activated. Necroptosis can activate dendritic cells by releasing DAMPs and a variety of immunomodulatory cytokines, and induce a strong immune response. Severe cases of COVID-19 are associated with hyperinflammation, also known as cytokine storm syndrome. Many innate immune cells, such as dendritic cells, natural killer (NK) cells, and adaptive immune cells, including B and T cells, are involved in cytokine storm syndrome. When the virus invades the body, it stimulates immune cells to release inflammatory cytokines. Inflammatory cytokines produced by immune cells, such as IL-1, IL-6, IL-12, IL-18, TNF, and INF-γ, play a central role in the cytokine storm [51]. Elevated levels of inflammatory cytokines, such as IL-4, IL-2, IL-6, IL-7, IL-10, MCP-1, TNF-α, and IFN-γ, were also observed in patients with COVID-19 [52, 53]. Inflammatory factors TNF-α and IFN-γ can induce necroptosis. Overactivation of intracellular MLKL and phosphorylation of RIPK1 after TNF-α and IFN-γ stimulation were observed [22]. TNF-α is involved in necroptosis driven by RIPK1, RIPK3, and Caspase-8 [54] and leads to phosphorylation of MLKL and then initiation of DAMPs [55]. DAMPs further stimulate downstream TLR signaling pathways, ultimately causing cytokine storm syndrome (Fig. 1). It has been reported that RIPK1, RIPK3, and MLKL are related to the severity of COVID-19 disease, and the levels of three regulatory factors in plasma of severe patients continue to increase [28]. At the same time, TLRs was also found in the blood and lungs of severe COVID-19 patients [56]. Current studies suggest that necroptosis is largely involved in the formation of cytokine storm syndrome in patients with COVID-19. Meanwhile, overactivation of necroptosis and cytokine storm syndrome may be associated with pneumonia severity.

A large number of compounds and agents have been found to control necroptosis and show significant anti-inflammatory effects, which may become therapeutic drugs for the treatment of COVID-19. However, most studies investigating the therapeutics targeting necroptosis are based on in vitro experiments and/ or animal models; thus, the feasibility of the clinical use of these necroptosis inhibitors still needs to be assessed in vivo and clinical trials.

Cytokine storms are the primary means of a series of pathological processes triggered by COVID-19. Therefore, anti-inflammatory drugs are first considered as the primary means of treatment for COVID-19 infection [57]. The glucocorticoids, such as dexamethasone and hydrocortisone, have first been noticed by scholars. Tomazini et al. observed that intravenous dexamethasone plus standard care increased the number of days alive and free of mechanical ventilation over 28 days [58]. Subsequent studies have shown that 10-day treatment with dexamethasone can effectively reduce the 28-day mortality of COVID-19 patients [59]. In addition, non-steroidal anti-inflammatory drugs (NSAIDs) also have to be mentioned. Among hospitalized patients with COVID-19, aspirin can slightly increase the rate of discharge within 28 days, but had no significant effect on 28-day mortality or disease progression [60]. From the results mentioned above, using conventional anti-inflammatory drugs for the treatment of COVID-19 is very limited. At the same time, elderly patients with cardiovascular disease or rheumatoid disease have been taking anti-inflammatory drugs, such as aspirin for a long time [61], and there is no relevant data to show the COVID-19 infection rate of this group is different from that of other groups. In addition, due to side effects, such as gastrointestinal, renal, and cardiovascular complications, the use of these drugs still needs to be cautious. Specific immunotherapy is also seen as an effective way to mitigate the progression of COVID-19. However, anti-interleukin drugs targeting IL-1 and IL-6 cannot shorten the treatment time of COVID-19 [62]. Subsequent studies have suggested that anti-IL-6 receptor antibody tocilizumab may be effective in patients with moderate to severe COVID-19, but it is not recommended as routine treatment [63]. The use of biologics can only inhibit the effects of specific inflammatory factors, and the effect is not good for cytokine storm syndrome in which multiple inflammatory factors are involved. Compared with the above drugs, necroptosis inhibitors can effectively inhibit systemic inflammatory response caused by programmed necroptosis /DAMPs and prevent the expansion of inflammation. In addition, the fact that patients can benefit from necroptosis inhibitors does not mean that they will treat all cases. The treatment of COVID-19 should still be a combination of prevention and treatment.

In addition to necroptosis, other types of programmed cell death, such as apoptosis and pyroptosis, are also involved in the pathogenesis of COVID-19. The inflammatory response is accompanied by excessive apoptosis of cells engaged in inflammation. Apoptosis could be a strategy for the resolution of inflammatory response [64]. Pyroptosis is an inflammation-dependent type of programmed cell death, which is mediated by inflammasomes [65]. Pyroptosis is a type of regulated cell death that it is mainly involved in proinflammatory events [66]. The main role of apoptosis and pyroptosis in COVID-19 has not been fully elucidated, and whether pathway inhibitors have an effect on the treatment of COVID-19 needs further research.

We focused on the relationship between COVID-19, necroptosis, and cytokine storms, as well as the potential of necroptosis inhibitors for treating COVID-19. Based on the important role of cytokine storm in the immunopathology of COVID-19, we also cannot deny the role of other factors such as NF-κB and MAPK in the regulation of necroptosis [67]. Current studies mainly focus on the direct impact of RIPK1 and other molecules on COVID-19, while the role of other molecules in the body in changing the progression of COVID-19 by influencing necroptosis remains to be further studied. In addition, the impact of necrosis inhibitors on COVID-19 to varying degrees remains to be further clarified. And the immune responses that may play a dominant role in the various stages of COVID-19 are unknown, so it is only prudent to speculate that necroptosis inhibitors may be beneficial in severely COVID-19 patients.

Collectively, necroptosis, the form of cell death that can trigger inflammatory responses by releasing inflammatory cytokines, may play a part in the pathogenesis and severity of COVID-19. Therefore, the necroptosis inhibitor is expected to become a promising therapeutic drug for the treatment of COVID-19. However, the effect of necroptosis inhibitor in the treatment of COVID-19 needs more clinical trial verification.

## Data Availability

The datasets used and/or analyzed during the present study are available from the corresponding author on reasonable request.

## Abbreviations

ARDS: Acute respiratory distress syndrome
BOAs: Benzoxazepinone
CCD: Coiled-coil domain
CDC37: Cell division cycle 37
CASP8: Caspase-8
cIAP1: Cellular inhibitor of apoptosis protein 1
COVID-19: 2019 Novel Coronavirus Disease
CSS: Cytokine storm syndromes
CYLDL: Cylindromatosis
DAMPs: Damage-associated molecular patterns
FADD: Fas-associated protein with death domain
HSP90: Heat shock protein 90
IDO: Indoleamine-2,3-dioxygenase
IFN-γ: Gamma interferon activation factor
IL-6: Interleukin-6
iNOs: Inducible NO synthase
IRF1: Interferon regulatory factor 1
KA: Kongensin A
MAPK: Mitogen-activated protein kinase
MLKL: Mixed lineage kinase domain-like protein
Nec-1 s: Necrostatin-1 s
NF-кB: Nuclear factor-kappa B
NK: Natural killer
NO: Nitric oxide
NSA: Necrosulfonamide
NSAIDs: Non-steroidal anti-inflammatory drugs
PK: Pharmacokinetics
RHD: Receptor homology domain
RIPK1: Receptor-interacting protein kinase 1
SARS-CoV-2: SARS coronavirus 2
SNX-5422: PF-04929113
STAT1: Signal transducers and activators of transcription 1
TLRs: Toll-like receptors
TNF-α: Tumor necrosis factor-alpha
TRADD: TNFR-associated death domain
TRAF2: TNFR-associated factor 2
VOC: Variants of concern
VOHC: Variants of high consequence
VOI: Variants of interest
17-AAG: Tanespimycin/CP 127374
